# Cadaveric Study on the Morphology and Morphometry of Heart Papillary Muscles

**DOI:** 10.7759/cureus.22722

**Published:** 2022-02-28

**Authors:** Pooja Bhadoria, Kanchan Bisht, Brijendra Singh, Vandana Tiwari

**Affiliations:** 1 Anatomy, All India Institute of Medical Sciences, Rishikesh, IND; 2 Anatomy, All India Institute of Medical Sciences, Bilaspur, IND

**Keywords:** chordae tendineae, cadaveric study, papillary muscles, cusp, valve

## Abstract

Introduction

A normal atrioventricular valve complex of the heart consists of the atrioventricular (A-V) ring, cusps, chordae tendineae, and papillary muscles. The right ventricle contains three while the left ventricle contains only two papillary muscles, which are named according to their location. A thorough understanding of the normal anatomy as well as possible variations can help surgeons in various corrective surgeries involving papillary muscles.

Material & methods

The study included 50 formalin-preserved hearts procured from human cadavers of unknown age and cause of death. The number of papillary muscles along with their shape, size, and pattern were noted separately for each ventricle. Data were analyzed using SPSS Version 21.0 (IBM Corp., Armonk, NY).

Results

The left and right ventricles contained two and three papillary muscles, respectively, in all the hearts. In the right ventricles, conical shape and the single base and divided apex (SBDA) pattern were found to be most prevalent. Anterior papillary muscles exhibited the mean length of 12.71±3.81 and 16.41±4.33 in the right and left ventricles, respectively. Similarly, posterior papillary muscles exhibited a mean length of 12.40±3.03 and 14.64±3.92 in the right and left ventricles, respectively. Both differences were found to be statistically significant

Conclusion

For the appropriate functioning of valves, both anatomical and mechanical coherence of the papillary muscles is required. A very keen understanding of this valvular complex is thus essential for anatomists, physiologists, and cardiologists to deal with normal as well as pathological valvular conditions.

## Introduction

A normal atrioventricular (A-V) valve complex of the heart consists of the A-V ring, cusps, chordae tendineae, and papillary muscles [1]. Chordae tendineae are fibrous structures connecting the apex of papillary muscles with the cusps of the atrioventricular valve [2]. The attachment is such that the anterolateral muscle controls the anterolateral half while the posterolateral muscle controls the posterolateral half of the atrioventricular valve [3]. The chordae tendineae attached to each papillary muscle in ventricles varies from two to 20 in number. When all the chordae tendineae in a left ventricle are connected to only one papillary muscle, it is referred to as a parachute mitral valve. It is often associated with mitral regurgitation and stenosis [4].

The papillary muscles are pillar-like muscles in the ventricular cavity. The anterior or anterolateral papillary muscle emerges from the sternocostal wall, the posterior or posteromedial muscle from the diaphragmatic wall of the ventricle, and the smallest one, the septal muscle, emerges from the inter-ventricular septum. During the systolic phase of the cardiac cycle, with ventricular contraction, the papillary muscles also contract simultaneously, making the chordae tendineae taut and thus preventing the prolapse of atrioventricular valves [4].

In the right ventricle, papillary muscles are three in number and are named according to their positions - anterior, posterior, and septal, while in the left ventricle, they are only two in number - anterior and posterior [1]. Due to higher blood volume in the left side of the heart and more workload for the left ventricle, the left-sided papillary muscles are believed to be stronger than those of the right ventricle [5]. The left ventricle usually contains two papillary muscles as mentioned in most anatomy books [6]. However, the presence of extra-papillary muscles has also been documented in some previous studies. An extra-papillary muscle is significant, as it might be sometimes mistaken for mural thrombi during a cardiac investigation, such as echocardiography, especially if the left ventricle is infarcted in that area [7].

Among the various papillary muscles, the anterior one is found to be the largest. The muscle of Lancisi is the most superior and largest of the small septal papillary muscles [8]. However, this nomenclature doesn’t hold true if the short axis of cardiac mass is analyzed in the left anterior oblique projection. This way, the muscles were found to be located inferoseptally and superolaterally and not “posteromedially” and “anterolaterally” [9].

The direction of papillary muscles makes a right angle with the direction of the atrioventricular ring, which is a mechanical advantage. They also prevent the bidirectional flow of blood in the heart by bringing about proper closure of the atrioventricular valves [10]. The heart is chiefly supplied by the right and left coronary arteries. With the maximum number of people being right coronary dominant, the right coronary artery supplies most of the structures in the heart, including the posteromedial papillary muscle [11].

Papillary muscles are supplied by deep-lying Purkinje fibers in the endocardium. Damage to papillary muscles or chordae tendineae can lead to papillary muscle dysfunction. Owing to its subendocardial location and blood supply from terminal branches of coronary arteries, the papillary muscles are prone to ischemia [2].

Papillary muscles in the left ventricle are believed to be sensitive indicators of myocardial ischemia since they are the last part of the heart receiving blood supply from coronary arteries [12]. This was also mentioned by Waller et al. in their study on the anatomy of the heart [7].

Patients with congenital variations in the atrioventricular complex are more prone to mechanical trauma, leading to valvular lesions in later life [13]. For performing various corrective surgeries involving the papillary muscles, such as resection or repositioning, the surgeon must have a keen knowledge of possible alterations in the usual anatomy [6].

The gradual delamination of the ventricular wall is responsible for the embryological development of heart muscles. Its incomplete delamination might lead to distortion in the external appearance of papillary muscles [14]. The interior of the heart is specially adapted to maintain the rapid conduction of cardiac impulses [15]. The left ventricular wall is almost three times thicker than the right one. Due to its lower mass, the right ventricle has lesser oxygen demand compared to the left ventricle [16].

The left atrioventricular or mitral valve apparatus has been observed to be specific for everyone just like fingerprints. Hence, for better post-op results, a surgeon should try to preserve the patient’s own papillary muscles. In fact, the homograft mitral valve has also been used for replacing the right-sided tricuspid valve with fair results [17].

## Materials and methods

The study was performed on 50 formalin-preserved hearts procured from human cadavers of unknown age and cause of death. Consent was not required for the same, as the donated bodies for teaching and research purposes were used after proper formalities.

The hearts were washed properly both externally and internally for residual clots or other debris. Proper incisions were given with the help of a scalpel knife and the interior of the heart was exposed adequately to visualize the papillary muscles.

The number of papillary muscles in each ventricle was noted along with the shape and pattern of each of them. The length (mm) and breadth (mm) of each papillary muscle were measured separately for the left and right ventricles with the help of Vernier calipers. The length was measured from the attachment of the base of the papillary muscle at the ventricular wall to the apex while breadth was measured as the width of the muscle at the origin from the ventricular wall.

Data were analyzed using SPSS Version 21.0 (IBM Corp., Armonk, NY). The chi-square test was used to compare proportional data (numbers and percentages). Mean values between left and right sides were compared using the paired t-test. A p-value of less than 0.05 indicated a statistically significant association.

## Results

In the present study, the number of papillary muscles in the left and right ventricles was constantly found to be two and three, respectively, in all the hearts.

**Figure 1:**
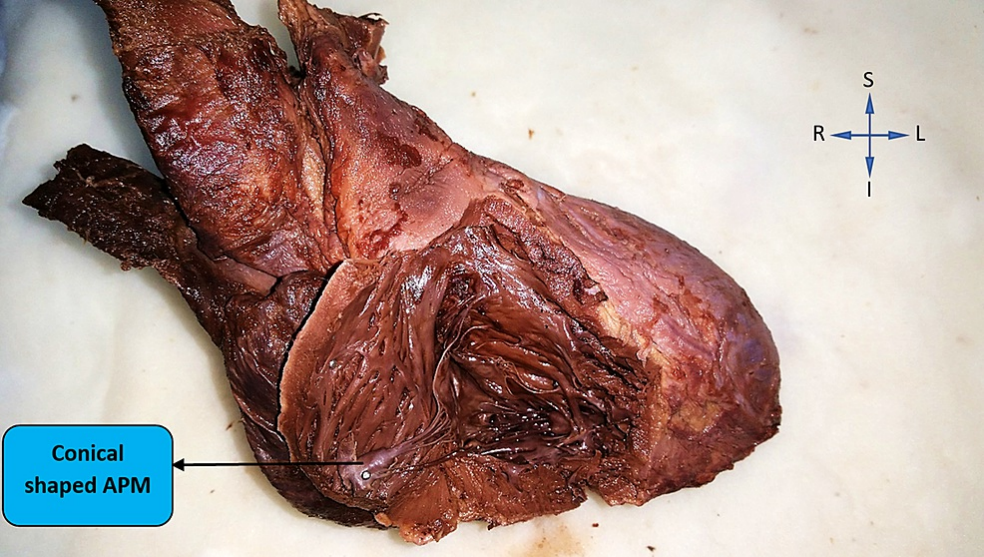
Conical-shaped anterior papillary muscle (APM) in the right ventricle

While observing the shape of the anterior papillary muscle in right ventricles, conical shape was seen in the majority of cases (42% (21)), pyramidal shape in 34% (17) cases, fan shape in 18% (9) cases, and broad apexed in 6% (3) cases. Figure 1 represents a right ventricle with conical-shaped anterior papillary muscles.

**Figure 2:**
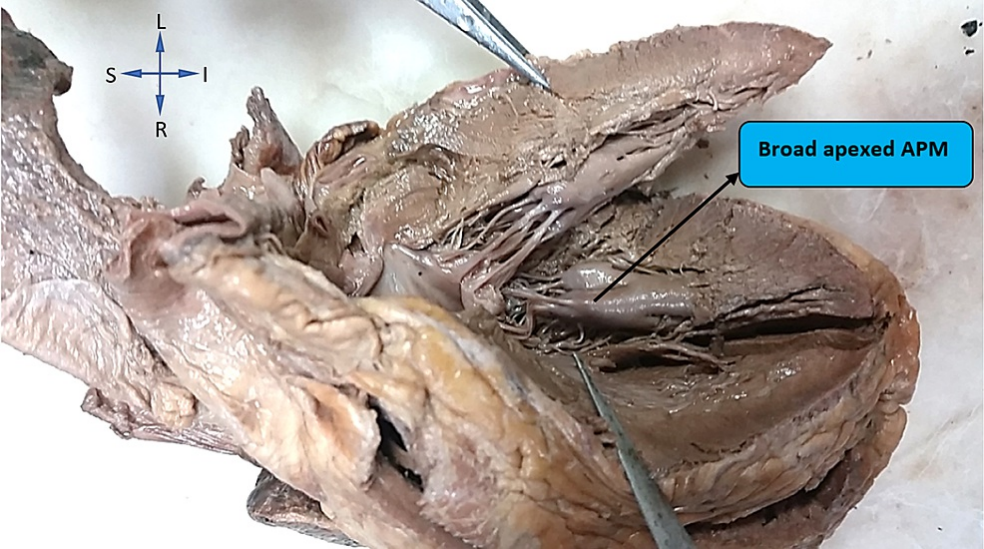
Showing broad apex anterior papillary muscle (APM) in the left ventricle

In the left ventricle, the shape of the anterior papillary muscle was found to be broad apexed in 32% (16) hearts, pyramidal shape in 30% (15) hearts, fan shape in 20% (10), and conical in 18% (9) hearts. However, this difference in shape of the anterior papillary muscles in the right and left ventricles was not statistically significant (p=0.003). Figure 2 represents a left ventricle with broad-apexed anterior papillary muscle.

**Figure 3:**
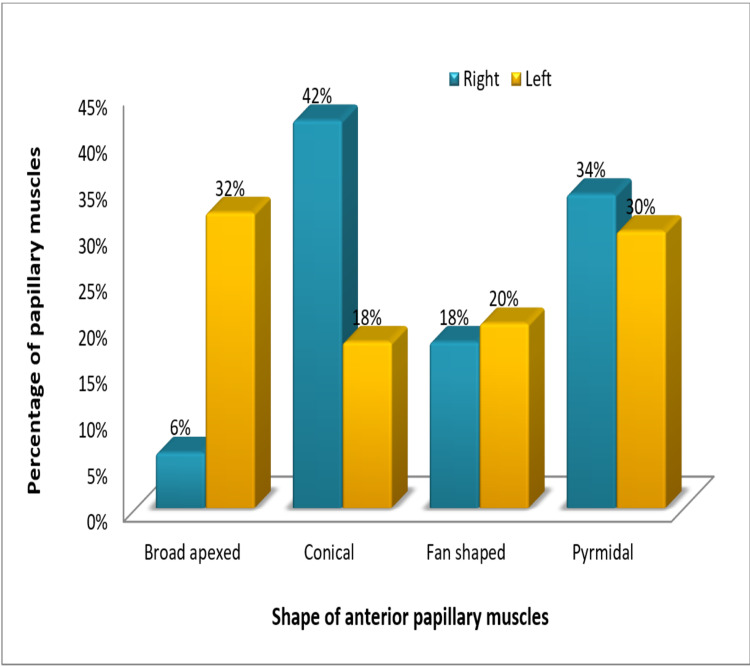
Comparison between the shape of anterior papillary muscle (APM) in the right and left ventricles

Figure 3 represents a comparison between the shape of anterior papillary muscles in the right and left ventricles.

**Figure 4:**
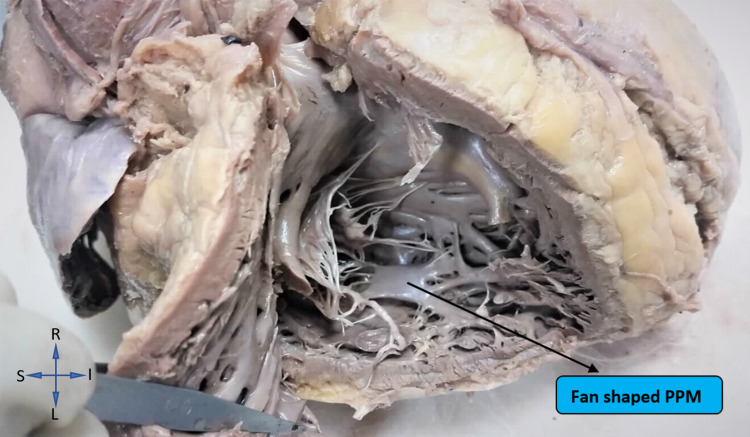
Showing fan-shaped posterior papillary muscle of the left ventricle

The shape of the posterior papillary muscle in the right ventricle was observed to be conical in 36% (18) cases; fan shaped in 24% (12), and broad apexed and pyramidal in 20% (10) cases each. While among the left ventricles, the shape of the posterior papillary muscle was seen to be fan-shaped in the majority of cases (38% (19)), as represented by Figure 4.

**Figure 5:**
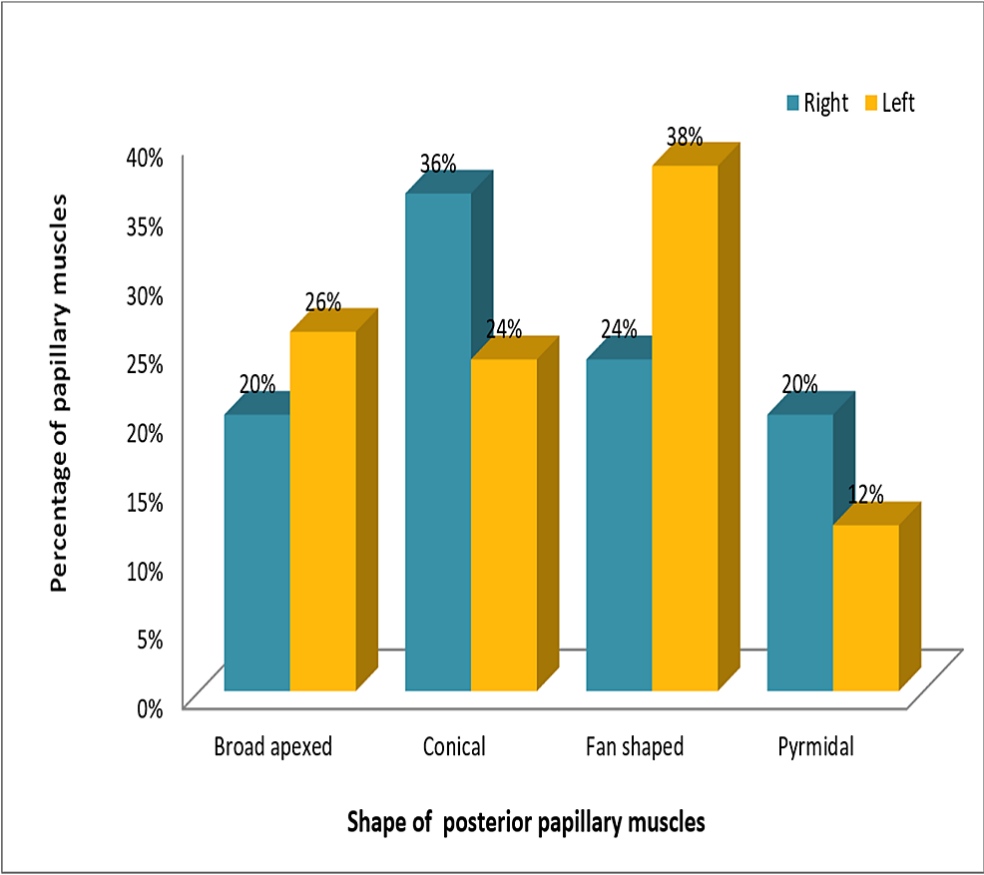
Comparison between the shape of the posterior papillary muscles in the right and left ventricles

Among the remaining left ventricles, the broad-apexed shape was found in 26% (13) hearts, conical in 24% (12) hearts, and pyramidal in 12% (6) hearts. Again, this difference in the shape of the posterior papillary muscles of the right and left ventricles was not significant statistically (p=0.243). Figure 5 represents a comparison between the shape of the posterior papillary muscles in the right and left ventricles.

**Figure 6:**
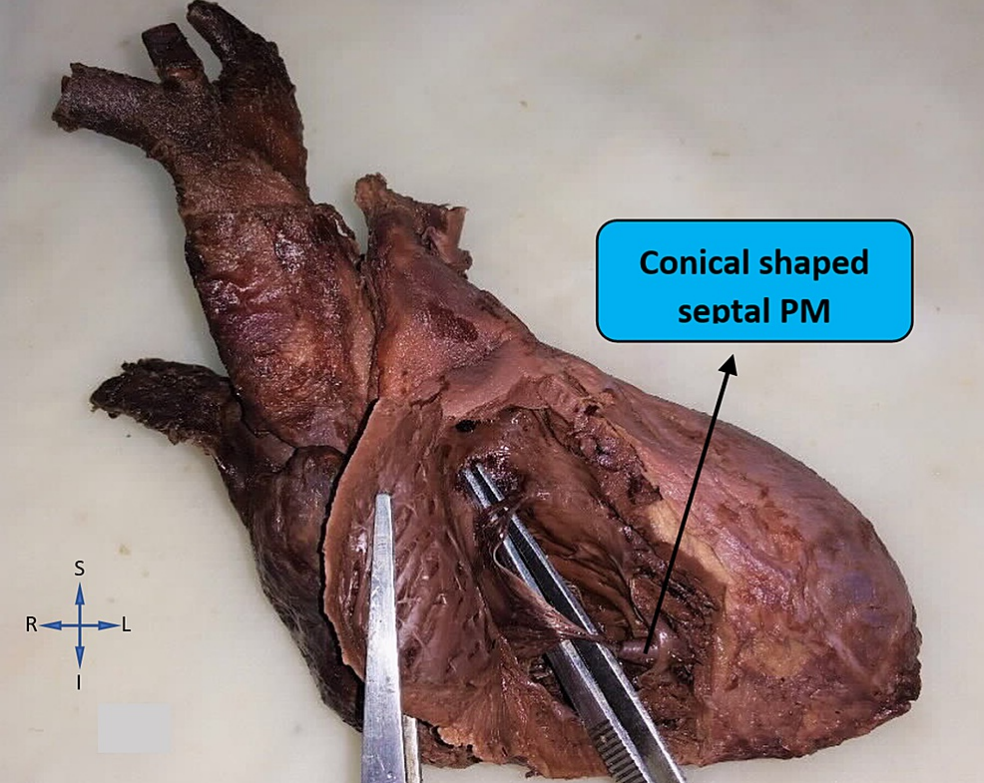
Conical-shaped septal papillary muscle in the right ventricle

The shape of the septal papillary muscle (SP), which was found only in the right ventricles, was uniformly found to be conical in all the 50 (100%) hearts included in our study. Figure 6 represents a right ventricle with conical-shaped septal papillary muscles.

**Figure 7:**
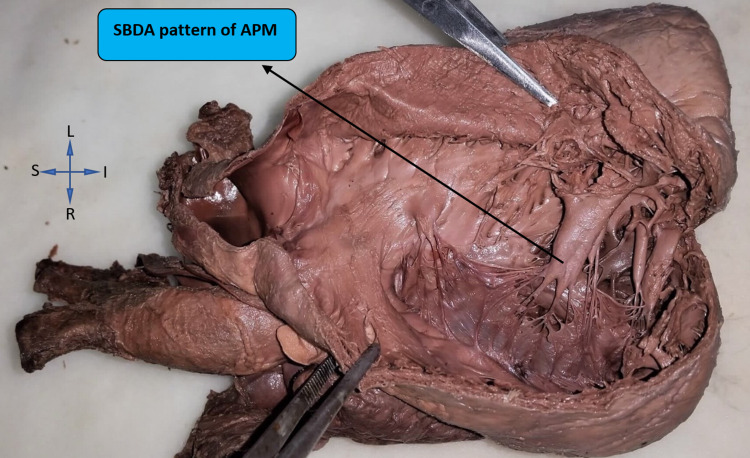
The most common type of anterior papillary muscle (APM) i.e. single base and divided apex (SBDA) in the right ventricle

The pattern of each papillary muscle was noted carefully. Based on our observation and previous literature, we classified the pattern as base attached to a large bridge (BL), long papillary muscles (LP), perforated papillary muscles (PPs), single base and divided apex (SBDA), single base fused apex (SBFA), and small projections of SPs. In the right ventricle, the pattern of anterior papillary muscle was seen as the SBDA type in 26% (13), BL and LP types each in 22% (11) hearts, SBFA type in 16% (8) hearts, and PP type in 14% (7) hearts. Figure 7 represents a right ventricle with the most common pattern (SBDA) of anterior papillary muscles.

**Figure 8:**
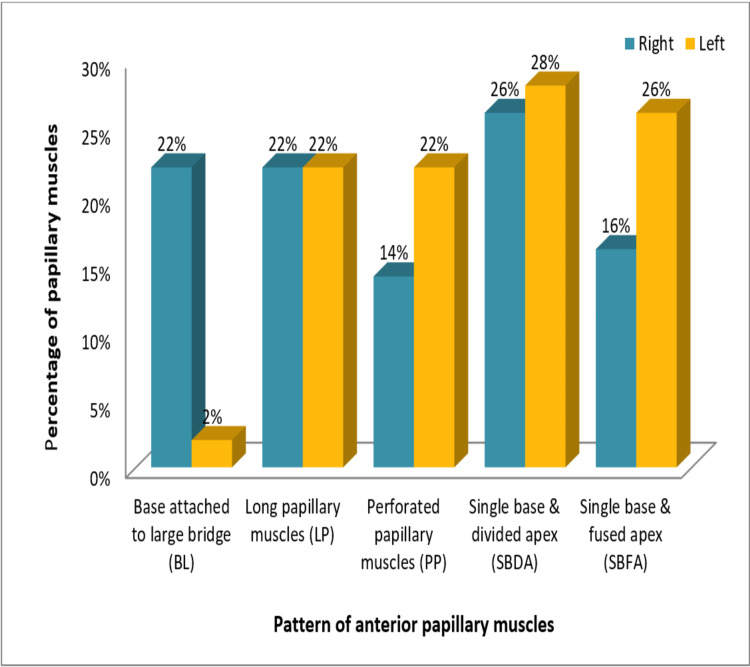
Comparison between the pattern of anterior papillary muscles (APMs) in the right and left ventricles

The left ventricles revealed the SBDA pattern in 28% (14) hearts, the SBFA pattern in 26% (13), LP and PP patterns each in 22% (11) hearts, and the BL pattern in only 2% (1) hearts in our study. The SP pattern was not found in the anterior papillary muscles of either ventricle. The comparison between the pattern of anterior papillary muscles in the right and left ventricles is represented in Figure 8.

**Figure 9:**
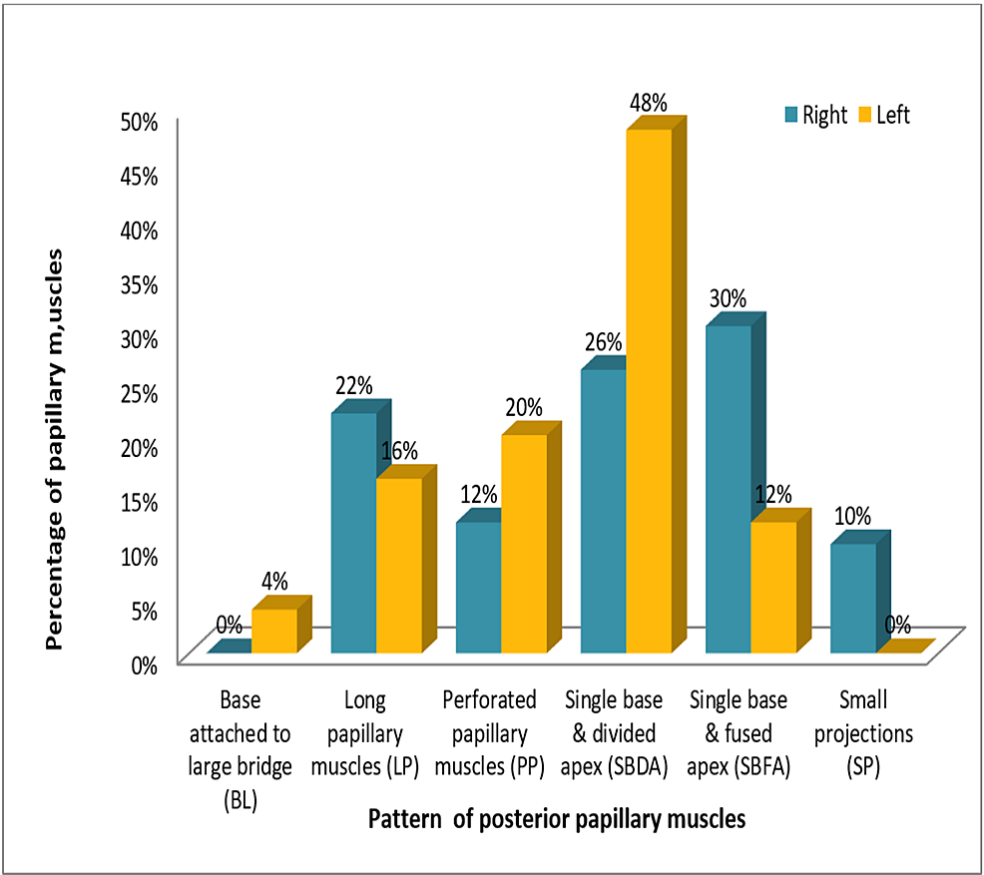
Comparison between the pattern of posterior papillary muscles in the right and left ventricles

The pattern of posterior papillary muscles in the right ventricle showed the SBFA type in 30% (15) cases, the SBDA type in 26% (13) cases, the LP type in 22% (11) cases, and the PP type in 12% (6) of total cases. The BL pattern was seen missing among posterior papillary muscles of the right ventricles of hearts in this study. However, the SP pattern was additionally seen in these muscles on the right side in 10% (5) cases. The posterior papillary muscles of left ventricles in our study were of the SBDA variety in 48% (24) hearts, PP in 20% (10), LP in 16% (8), SBFA in 12% (6), and BL in 4% (2) of total hearts. Figure 9 represents the comparison between the pattern of posterior papillary muscles in the right and left ventricles.

The pattern of septal papillary muscles, present only in right ventricles, was uniformly seen to be of the SP variety in all 50 (100%) hearts in our study. The differences in the patterns of anterior and posterior papillary muscles of the right and left ventricles were not found to be statistically significant (p=0.033 and p=0.008, respectively). A statistically significant difference (t=8.664; p<0.001) was observed between the mean length of anterior papillary muscles in the right (12.71±3.81) and left ventricles (16.41±4.33). Similarly, a statistically significant difference (t=4.912; p<0.001) was observed between the mean length of posterior papillary muscles in the right (12.40±3.03) and left ventricles (14.64±3.92).

The mean for the width of anterior papillary muscles in the right and left ventricles was recorded as 6.92±2.55 and 7.98±2.53, respectively. While for posterior papillary muscles, the mean of width in the right and left ventricles was recorded as 7.46±2.75 and 8.44±3.19, respectively. Both these comparisons were not significant statistically (p=0.014 and p=0.013, respectively). The mean for the length and width of septal papillary muscles, present only in right ventricles, was found to be 1.67±0.48 and 1.08±0.72, respectively. A comparison between the dimensions of papillary muscles in the right and left ventricles is represented in Table 1.

**Table 1:** Comparison between the dimensions of papillary muscles in right and left ventricles

S. No.	Parameter		Right	Left	Statistical difference
1.	Length (Anterior)		12.71±3.81	16.41±4.33	t=8.66; p<0.001
2.	Width (Anterior)		6.92±2.55	7.98±2.53	t=2.55; p=0.014
3.	Length (Posterior)		12.40±3.03	14.64±3.92	t=4.91; p<0.001
4.	Width (Posterior)		7.46±2.75	8.44±3.19	t=2.57; p=0.013
5.	Septal length		1,67±0.48		-
6.	Septal width		1.08±0.72		-

## Discussion

For performing various corrective surgeries involving papillary muscles, such as resection or repositioning, the surgeon must have a keen knowledge of normal anatomy as well as possible variations of papillary muscles [6].

In our study, the right and left ventricles of all 50 hearts were found to contain three and two papillary muscles, respectively. Most anatomy textbooks indicate the existence of two papillary muscles in the left ventricle, which is well in accordance with the findings of our study as well as the one conducted by Shree et al. [12]. In rare situations, Oosthoek et al. and Madu et al. reported an extra papillary muscle lying near to the apex [18-19]. Lakhanpal et al., in their research work on papillary muscles of the bicuspid valve in the Central Indian population, reported the presence of accessory anterior papillary muscles in 31% and accessory posterior papillary muscles in 25% of hearts, respectively [7].

Various studies carried out on right ventricles of different populations by Kumar et al., Nigri et al., Balachandra et al., and Wafae et al. demonstrated the presence of anterior and posterior papillary muscles in all instances, which is consistent with the findings in our study [20-23]. However, Begum et al., in their study on right ventricles, reported the absence of anterior papillary muscles in 8% of cases [24]. Similarly, the absence of posterior papillary muscles was reported by Harsha et al., Begum et al., and Gerola et al. in almost 1%, 40%, and 16% of cases, respectively, in their studies [1,24-25].

In their study on right ventricles, Nigri et al. found that septal papillary muscles were rudimentary, often missing, or extremely tiny in size with almost 21.5% of hearts lacking septal papillary muscles in right ventricles [21]. Similar findings were reported by Harsha et al., Kumar et al., and Begum et al. when septal papillary muscles were absent in 4.2%, 38.88%, and 24% cases, respectively, in their studies [1,20,24]. In our study, all the heart specimens (100%) revealed the presence of septal papillary muscles, which was in accordance with the studies conducted by Balchandra et al., Wafae et al., and Gerola et al. [22-23,25]. Table 2 represents the comparison between different studies indicating the presence or absence of papillary muscles in the right ventricle.

**Table 2:** Comparison showing the presence of papillary muscles in right ventricles in different studies APM: anterior papillary muscle; PPM: posterior papillary muscle; SPM: septal papillary muscle

S.No.	Studies	No. of cases	% of APM (Right)	% of PPM (Right)	% of SPM (Right)
1.	Harsha et al. [1]	96	100	98.95	95.8
2.	Balchandra et al. [22]	96	100	100	100
3.	Wafae et al. [23]	50	100	100	100
4.	Begum et al. [24]	50	92	60	76
5.	Gerola et al. [25]	50	100	84	100
6.	Present study	50	100	100	100

The shape of the papillary muscles has an important role in smooth blood flow. In most textbooks, papillary muscles are characterized as conical in form, which is best adapted to facilitate cardiovascular physiology by obstructing the blood flow as little as possible [12]. In our study, the most common shape we came across among papillary muscles of right ventricles was conical: 42% in anterior, 36% in posterior, and 100% in septal papillary muscles. This was in consonance with the studies conducted by Hospatana et al. and Nigri et al. [13,21]. In the study conducted by Hospatana et al., cone-shaped papillary muscles were observed in the majority of the cases (87%), whereas the flat-topped shape was observed in only 13% of cases in the right ventricle. In the left ventricle, all the papillary muscles were found to be cone-shaped (100%) [13]. Nigri et al., in their study on right ventricles, observed the conical shape to be the most common in anterior, posterior, as well as septal muscles (57%, 74%, and 86.4%, respectively) as believed by most anatomy textbooks [21].

Among the papillary muscles of left ventricles, the most common shape observed in our study was broad-apexed (32%) in anterior muscles and fan-shaped in posterior papillary muscles (38%). This was in consonance with the study performed by Gunnal et al. on papillary muscles of left ventricles. The specimens in their study exhibited the broad-apexed shape as the most common type (50.48%), followed by the conical shape (45.51%) [6].

Our findings were a little different from those obtained by Saha et al. in their study on right ventricles. The most common shape they came across among anterior, as well as posterior papillary, muscles was flat top (51.5% and 88.23%, respectively) [10]. However, in septal muscles, the maximum specimens showed the conical shape (90.62%), followed by the flat-top shape (9.37%).

Our study revealed that separate base and divided apex (SBDA) was the most common pattern among the anterior papillary muscles of right as well as left sides (26% and 28%, respectively) while among the posterior papillary muscles, separate base and fused apex (SBFA) was the most common pattern observed on the right side and SBDA on the left side. This finding was somewhat closer to that obtained by Gunnal et al. who demonstrated the SBFA and SBDA pattern to be the most common pattern (21.55% each) of papillary muscles in left ventricles in their study [6].

Various studies have been carried out till now, measuring the dimensions of papillary muscles. Atrioventricular regurgitation occurs when the papillary muscles are congenitally elongated, and this is caused by faulty valve closure [10]. The mean length calculated for anterior papillary muscles in the right and left ventricles came out to be 12.71±3.81 and 16.41±4.33, respectively, in our study. Similarly, the mean length calculated for posterior papillary muscles in the right and left ventricles was 12.40±3.03 and 14.64±3.92, respectively. The mean of septal papillary muscles measured 1,.67±0.48. So, we concluded that anterior papillary muscles are the longest and septal the smallest among all the papillary muscles, irrespective of the right or left side, and that the papillary muscles in the left ventricles are comparatively longer than their counterparts on the right side. These findings were supported by the research work done by Hospatana et al., Nigri et al., Beguma et al., and Farzana et al. in different parts of the globe [13,21,24,26]. Hospatana et al., in their study, found that the papillary muscles in the left ventricle were longer than those on the right [13]. The anterior and septal papillary muscles, as demonstrated by Begum et al. in their study on right ventricles, were the longest and smallest, respectively [24]. The study conducted by Nigri et al. was also in agreement with these findings where the longest muscles were anterior (19.16 mm) while the shortest were again septal papillary muscles (5.59 mm) [21]. Farzana et al., in their study, also observed that the length of anterior papillary muscles was more compared to other papillary muscles in both the right as well as left ventricles. An additional finding suggested by this study was that with advancing age, the average length of all the papillary muscles also keeps increasing [26].

Our study calculated the mean width of anterior papillary muscles in the right and left ventricles as 6.92±2.55 and 7.98±2.53, respectively, while for posterior papillary muscles, the mean width in the right and left ventricles was 7.46±2.75 and 8.44±3.19, respectively. So, we concluded that the width of papillary muscles in the left ventricles was more compared to the right ventricles in our study. However, we could not find much data in this regard to compare with our findings. The comparative analysis showing measurement of papillary muscles in right ventricles in different studies is represented in Table 3.

**Table 3:** Comparison showing the measurement of papillary muscles in right ventricles in different studies APM: anterior papillary muscle; PPM: posterior papillary muscle; SPM: septal papillary muscle

S.No.	Studies	No. of cases	Mean length (Right)			Mean width (Right)		
			APM	PPM	SPM	APM	PPM	SPM
1.	Harsha et al. [1]	96	1.49±0.44	1.05±0.37	0.7±0.22	0.82±0.21	0.63±0.17	0.48±0.16
2.	Priya et al. [2]	56	12.65±3.58	-	-	4.04±1.53	-	-
3.	Saha et al. [10]	52	2.19±0.59	1.39±0.63	0.95±0.38	0.76±0.26	0.67±0.43	0.59±0.09
4.	Hospatana et al. [13]	15	1.30±0.40	0.98±0.40	0.55±0.20	-	-	-
5.	Kumar et al. [20]	36	1.54±0.27	0.97±0.25	0.21±0.1	0.42±0.11	0.30±0.09	0.11±0.01
6.	Nigri et al. [21]	79	1.9	1.1	0.56	-	-	-
7.	Gerola et al. [25]	50	0.9±0.2	0.9±0.2	1.1±0.3	1.2±0.3	0.7±0.2	1.20±0.3
8.	Farzana et al. [26]	80	1.60±0.25	1.37±0.34	0.81±0.35	-	-	-
9.	Present study	50	12.71±3.81	12.40±3.03	1.67±0.48	6.92±2.55	7.46±2.75	1.08±0.72

Due to insufficient data on the measurement of papillary muscles in the left ventricle, we could not compare our findings of left ventricles with previous works.

During our study, we did not classify the specimens based on sex, so cannot comment on sexual dimorphism in the case of papillary muscles of the human heart. Begum et al., in their study on right ventricles, could not find the difference between the total number of papillary muscles in males and females to be significant statistically [24]. Although no statistical difference was reported by Kumar et al. while comparing the shape or the total number of papillary muscles in males and females in their study, septal papillary muscles were found to be missing from right ventricles in most of the male cadaveric hearts [20].

Limitations of the study

The present study has a very small sample size and it was restricted to hearts that were procured from donated cadavers in the institute. This could limit the generalizability of our findings to a bigger population. So the authors would like to propose a bigger study in the future on a larger number of specimens for better representation of the findings.

## Conclusions

For the optimal functioning of cardiac valves, both anatomical and mechanical coherence of the papillary muscles is required. A thorough understanding of the normal anatomy, as well as possible variations of papillary muscles, is thus essential for the early diagnosis of many cardiac anomalies and can be helpful for surgeons in various corrective cardiac surgeries. This is also essential for anatomists, physiologists, and cardiologists to deal with normal as well as pathological valvular conditions.

Our study provides a morphometric understanding of these papillary muscles with the findings indicating that the left ventricle of the heart is equipped with larger muscles, probably owing to its heavier function in pumping more blood volume. Our study reported the existence of three and two papillary muscles in the right and left ventricles, respectively, as mentioned in standard textbooks. Single base and divided apex (SBDA) is the most common pattern observed in the papillary muscles of hearts in our study. We also concluded that the mean length and width of papillary muscles in left ventricles are more as compared to their counterparts on the right side, and, overall, the anterior papillary muscles are the longest of all papillary muscles.
